# Encapsulation of Therapeutic, Low-Molecular-Weight Chemokines Using a Single Emulsion, Microfluidic, Continuous Manufacturing Process

**DOI:** 10.3390/pharmaceutics17081056

**Published:** 2025-08-14

**Authors:** Julie A. Kobyra, Michael Pezzillo, Elizabeth R. Bentley, Stephen C. Balmert, Charles Sfeir, Steven R. Little

**Affiliations:** 1 Department of Bioengineering, University of Pittsburgh, Pittsburgh, PA 15260, USA; juk90@pitt.edu (J.A.K.); erb122@pitt.edu (E.R.B.); 2 Department of Chemical Engineering, University of Pittsburgh, Pittsburgh, PA 15260, USA; mjp179@pitt.edu; 3 Department of Dermatology, School of Medicine, University of Pittsburgh, Pittsburgh, PA 15213, USA; 4 Center for Craniofacial Regeneration, School of Dental Medicine, University of Pittsburgh, Pittsburgh, PA 15261, USA; 5 Department of Periodontics and Preventive Dentistry, School of Dental Medicine, University of Pittsburgh, Pittsburgh, PA 15261, USA; 6 Department of Oral and Craniofacial Sciences, School of Dental Medicine University of Pittsburgh, Pittsburgh, PA 15261, USA; 7 McGowan Institute for Regenerative Medicine, University of Pittsburgh, Pittsburgh, PA 15219, USA; 8 Department of Clinical and Translational Science, University of Pittsburgh, Pittsburgh, PA 15213, USA; 9 Department of Immunology, University of Pittsburgh, Pittsburgh, PA 15213, USA; 10 Department of Pharmaceutical Sciences, University of Pittsburgh, Pittsburgh, PA 15213, USA; 11 Department of Ophthalmology, University of Pittsburgh, Pittsburgh, PA 15213, USA

**Keywords:** biologics, biodegradable polymers, controlled release, drug delivery, microparticle, double and single emulsion, microfluidics

## Abstract

**Background/Objectives:** Controlled release systems, such as polymeric microparticles (MPs), have emerged as a promising solution to extend the bioavailability and reduce dosing frequency for biologic drugs; however, the formulation of these systems to encapsulate highly sensitive, hydrophilic biologic drugs within hydrophobic polymers remains a nontrivial task. Although scalable manufacturing and FDA approval of single emulsion processes encapsulating small molecules has been achieved, scaling more complex double emulsion processes to encapsulate hydrophilic biologics remains more challenging. **Methods**: Here, we demonstrate that two hydrophilic, low-molecular-weight, recombinant chemokines, CCL22 and CCL2, can be encapsulated in poly(lactic-co-glycolic acid) (PLGA) MPs using a single emulsion method where the proteins are dissolved in an organic solvent during formulation. **Results**: As expected, we observed some differences in release kinetics from single emulsion MPs compared to double emulsion MPs, which traditionally have been used to encapsulate proteins. Single emulsion MPs exhibited a substantially reduced initial burst. Importantly, protein released from single emulsion CCL22-MPs also retained biological activity, as determined by a cell-based functional assay. Decreasing particle size or changing the polymer end group from PLGA-COOH to PLGA-OH increased the initial burst from single emulsion MPs, demonstrating tunability of release kinetics for protein-loaded, single emulsion MPs. Finally, to improve scalability and enable more precise control over MP formulations, the single emulsion process was adapted to a microfluidic, continuous manufacturing system, and the resulting MPs were evaluated similarly. **Conclusions**: Altogether, this study demonstrates the feasibility of using a single emulsion encapsulation method for at least some protein biologics.

## 1. Introduction

Since the introduction of the first biologic-based therapeutic in 1982, the market for biologics has seen a steady increase [1]. For instance, in 2024 alone, 50 new drugs were approved by the FDA, 16 of which were biologics [2,3]. A common class of biologics are therapeutic proteins, consisting of monoclonal antibodies, cytokines, and growth factors, which are used for a variety of different applications, including the treatment of autoimmune diseases (e.g., rheumatoid arthritis) and cancers (e.g., leukemia) [3,4]. Biologics are commonly used for their specificity and limited toxicity compared to traditional small-molecule-based drugs. Although biologics are proving to be extremely powerful therapeutic options, there are several complications associated with their use. Therapeutic proteins typically have a complex structure and are sensitive to environmental conditions and processes (e.g., enzyme degradation), often resulting in a short half-life and low bioavailability [5]. As a result, biologics typically need to be administered frequently and/or in high doses to produce a therapeutic effect. However, frequent administration of high doses is often associated with poor patient compliance, increased costs, and unwanted side effects. As a result, research has focused on how to improve the half-life of therapeutic proteins to reduce dosing frequency without compromising efficacy or causing adverse effects.

One promising approach used to address these limitations is the use of drug delivery systems, such as polymer-based microparticles (MPs) [6,7]. MPs have been used often due to their versatility, as they can encapsulate a variety of different therapeutic agents ranging from small molecules to biologics, degrading at a defined rate in a local environment. Encapsulation of water-soluble biologics, such as therapeutic proteins, typically involves a double emulsion (DE) solvent evaporation method, which can reduce protein denaturation by protecting the protein in an aqueous phase [8,9,10,11]. An inner aqueous phase containing the therapeutic protein is added to an organic solvent containing a polymer (e.g., poly(lactic-co-glycolic) acid (PLGA)) and sonicated to form a primary water-in-oil (W/O) emulsion. The first emulsion is then added into an outer aqueous phase containing an emulsifying agent, which provides emulsion stability, and undergoes homogenization to generate the second oil-in-water (O/W) emulsion, yielding MPs after solvent evaporation. While this method is suitable for hydrophilic molecules like biologics, it is a complex, multi-step batch process, reducing translatability and making it more difficult to scale [12].

In contrast to a double emulsion process, an oil-in-water (O/W) single emulsion (SE) solvent evaporation method is much simpler and easier to scale [13]. Using this approach, the therapeutic agent (typically a hydrophobic small molecule) is added into the same phase as the polymer or dissolved in a different organic solvent, such as dimethyl sulfoxide (DMSO), which is then added to the polymer solution and homogenized to generate MPs [14]. Therapeutic proteins can be easily destabilized in organic solvents used with polymers. This destabilization can take the form of disruption of secondary structures (e.g., alpha helices), as well as tertiary structures [15,16,17]. Nonetheless, several groups have previously shown the feasibility of utilizing a similar approach, where a protein of interest was complexed to different surfactants and lyophilized prior to solvation in the polymer organic phase or DMSO [18,19,20,21]. The presence of a surfactant can stabilize the protein of interest in the solvent used. Limited studies have been conducted to investigate the feasibility of encapsulating biologics without the added presence of a stabilizing surfactant.

In this study, an SE technique was explored to encapsulate smaller, less complex proteins, such as chemokines. Chemokines are a type of immunomodulatory protein known to direct the migration and polarization of immune cells. Their sustained release is necessary to establish gradients that are required for cell recruitment [22]. Prior works by our group have encapsulated CCL22, which recruits regulatory T cells, and CCL2, which recruits macrophages via DE solvent evaporation techniques. Both CCL22 and CCL2-MPs were found to induce cell recruitment and provided therapeutic benefit in the context of inflammatory models [23,24,25,26]. Their previous application, as well as their limited secondary and tertiary structures, made them excellent candidates for encapsulation via an SE technique. To this end, CCL22 and CCL2 were encapsulated utilizing both the traditional DE batch process (using water as a solvent), as well as an SE batch process (using organic solvent), and both types of formulations were characterized and compared in regard to key formulation outcomes. Furthermore, to better enable translation, the SE batch process was then adapted into a continuous manufacturing process using a microfluidic system. MPs manufactured via the batch and continuous processes were compared, and differences in release kinetics were identified. Altogether, this study demonstrates that some proteins, including CCL22 and CCL2, can be encapsulated successfully via an SE technique without surfactant stabilization utilizing both batch and continuous processes.

## 2. Materials and Methods

### 2.1. Materials

Recombinant human CCL22 (rhCCL22) and recombinant human CCL2 (rhCCL2; Peprotech, Cranberry, NJ, USA) were purchased from Fisher Scientific. Bovine serum albumin (BSA) was purchased from Tocris Biosciences (Bristol, UK). Poly vinyl alcohol (PVA; MW ~25 kDa, 98% hydrolyzed) was purchased from Polysciences (Warrington, PA, USA). Poly(lactic-co-glycolic acid) hydroxyl-terminated (PLGA-OH; 50:50 lactic/glycolic acid, MW: 10 kDa) and acid-terminated (PLGA-COOH; 50:50 lactic/glycolic acid, MW: 10 kDa) were purchased from Nanosoft polymers (Winston-Salem, NC, USA). Sodium chloride (NaCl) was purchased from Fisher Scientific (Waltham, MA, USA). Enzyme linked immunosorbent assays (ELISAs) for rhCCL22 and rhCCL2 were obtained from R&D systems (Minneapolis, MN, USA).

### 2.2. Microparticle Fabrication via Homogenization (Batch Process)

#### 2.2.1. Double Emulsion

PLGA MPs were manufactured using a water-in-oil-in-water (W/O/W) double emulsion technique, as described previously [23,24,26]. All double emulsion MPs were manufactured using a 10 kDa PLGA. MPs were loaded with 25 μg of CCL22 or CCL2. The inner aqueous phase (200 μL), which also contained 10 mg/mL BSA and 15 mM NaCl, was directly added into PLGA dissolved in dichloromethane (DCM, 4 mL) and sonicated at an amplitude of 55% for 10 s to create the first water-in-oil emulsion (W/O). This emulsion was then poured into a solution of 2% polyvinyl alcohol (PVA) and homogenized at 3000 rpm for 1 min. The resulting water-in-oil-in-water (W/O/W) emulsion was then added to 1% PVA at room temperature and stirred for 3 h to allow for the DCM to evaporate. MPs were then collected and washed four times with deionized water, resuspended in 5 mL of deionized water, flash frozen, and lyophilized for 72 h. Following lyophilization, MPs were stored at −20 °C until further analysis.

#### 2.2.2. Single Emulsion

PLGA MPs loaded with CCL22 or CCL2 were made using an oil-in-water (O/W) single emulsion protocol, as described previously for the formulation of rapamycin-loaded MPs [27]. Single emulsion MPs were manufactured using 10 kDA PLGA that either was carboxylic acid (PLGA-COOH) or hydroxyl (PLGA-OH) capped. Briefly, 25 μg of lyophilized CCL22/CCL2 was dissolved in 200 μL of DMSO and mixed with 200 mg of PLGA dissolved in 4 mL DCM. This solution was immediately added into a solution of 2% PVA and homogenized for 1 min at either 3000 rpm for smaller MPs or 2000 rpm for larger MPs. The resulting emulsion was then added into 1% PVA at room temperature and stirred for 3 h to allow for the evaporation of DCM. MPs were collected and washed as previously described in Section 2.2.1.

### 2.3. Microparticle Fabrication via Microfluidics (Continuous Process)

The microfluidic system was set up and utilized as previously described [28]. Briefly, a microfluidic system (Dolomite, Royston, UK) was used to manufacture single emulsion CCL22-MPs and CCL2-MPs. As for the single emulsion batch process, 25 μg of CCL22 or CCL2 was reconstituted in 200 μL of DMSO and immediately mixed with 200 mg of polymer dissolved in 4 mL DCM, forming the organic phase. The organic phase and an outer aqueous phase of 2% PVA were pumped into separate channels of a 100 μm diameter flow-focusing glass microfluidic chip (Dolomite #3200433) using pressure pumps. Flow rates for both the aqueous phase (PVA) and organic phase (protein and PLGA in DCM) were monitored by flow sensors. The flow rate of the aqueous phase was set at 70 μL/min, while the flow rate of the organic phase was set to 7 μL/min to form droplets. Droplets were then collected for 3 h in a hardening bath containing 1% PVA while under stirring conditions (600 rpm). After 3 h, the mixture was further stirred for an additional 3 h to allow for the complete evaporation of DCM. MPs were then collected and washed with deionized water four times, flash frozen in liquid nitrogen, and lyophilized for 72 h. After lyophilization, particles were stored at −20 °C until further analysis.

### 2.4. Microparticle Characterization

Both single and double emulsion CCL22-MPs and CCL2-MPs were characterized to assess surface morphology, size distributions, and protein release kinetics. Size distributions were assessed via volume impedance measurements (Beckman Coulter Counter, Brea, CA, USA; *n* = 10,000 particles). Protein release was determined as previously described [23,24,25,26,27,29]. Briefly, 5–8 mg of CCL22 or CCL2-MPs were resuspended in microcentrifuge tubes containing 1 mL PBS with 1% bovine serum albumin (BSA), which were then placed on an end-over-end rotator at 37 °C. Release media with 1% BSA (10 g/L) is consistent with albumin concentrations in interstitial fluid [30]. To ensure there was no contamination of the release media, PBS with 1% BSA was sterile filtered, kept at 4 °C, and checked regularly for microbial growth. Every other day MPs were centrifuged, and supernatants were extracted then stored at −20 °C until an analysis via enzyme-linked immunosorbent assays (ELISAs). MPs were then resuspended in 1% BSA in PBS and placed back on the rotator. Surface characterization of MPs was conducted using scanning electron microscopy (SEM; Zeiss, Jena, Germany).

### 2.5. Biological Activity Testing

To generate samples for biological activity testing, 15 mg of microparticles were resuspended in 1% BSA in PBS and placed on a rotator at 37 °C. Following 14 days of incubation, microparticles were centrifuged, and the supernatant was removed and stored for later analysis. To assess biological activity, a Tango CCR4-bla U2OS cell-based assay (Waltman, MA, USA), was utilized. Briefly, Tango cells were plated 16–24 h prior to the start of the assay. The next day, 8 serial dilutions of fresh CCL22 were generated. For a negative control, CCL22 was denatured via exposure to 95 °C heat for 24 h, and serial dilutions were made accordingly. For microparticles, 8 serial dilutions were also generated. Serial dilutions of fresh, denatured, and released CCL22 were added into the wells and incubated at 37 °C for 5 h. Following a 5 h incubation, a substrate mixture was added into each well and the plate was left to incubate at room temperature protected from light for 2 h. At the end of the 2 h period, fluorescence readings were obtained. The response ratio between the blue and green channel was then calculated.

### 2.6. Fluorescent Protein Labeling

CCL22 (100 μg) was labeled with AF647 using an Alexa Fluor 647 microscale protein labeling kit (Thermo Fisher #A30009, Waltman, MA, USA) according to the manufacturer’s instructions. A 5:1 dye to protein molar ratio was used. Protein concentration and degree of labeling was determined using a DeNovix DS-11 Spectrophotometer (Wilmington, DE, USA). Protein was then flash frozen in liquid nitrogen and lyophilized for 48 h. After lyophilization, protein was stored at −80 °C.

### 2.7. Fluorescent Microparticle Fabrication

Fluorescent microparticles were manufactured using both a double and single emulsion technique as described in Section 2.2.1 and Section 2.2.2. CCL22 fluorescent microparticles were manufactured using only the carboxylic acid-terminated PLGA. MPs were utilized to more accurately assess protein loading.

### 2.8. Protein Loading Capacity

To determine the amount of protein loaded, fluorescent CCL22 MPs were utilized. Briefly, 5 mg of particles were dissolved in 700 μL of DMSO. The same fluorescent protein used for the manufacturing of MPs was used to generate a standard curve in DMSO. Samples were then plated on a 96-well solid black plate. Fluorescent readings were taken at an excitation wavelength of 640 nm and an emission wavelength of 680 nm. Total loaded protein was interpolated using the standard curve and reported per mass of MPs.

## 3. Results and Discussion

### 3.1. Double Emulsion Encapsulation of CCL22

**Figure 1:**
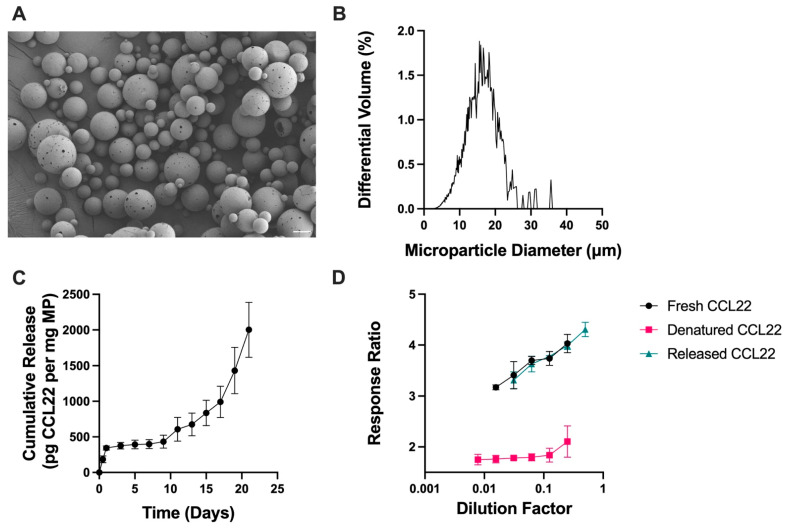
Characterization of double emulsion CCL22 microparticles. (**A**) Representative SEM image demonstrating the porous, spherical particles. (**B**) Size distribution as determined by volume impedance measurements (mean diameter 17.4 ± 7.0 μm). (**C**) Release kinetics of CCL22 over a 21-day period, as determined via in vitro release assay and ELISA. Data are mean ± SEM (*n* = 3). (**D**) Biological activity of released CCL22 compared to fresh and denatured CCL22 measured using a cell-based assay. Higher response ratio corresponds to greater bioactivity. Results are mean ± SD (*n* = 3).

CCL22-MPs were first manufactured using a lab-scale, homogenization-based, double emulsion (DE) solvent evaporation method, which is typically used to encapsulate hydrophilic proteins. CCL22-MPs formulated with 10 kDa MW PLGA-COOH using the DE method were characterized to assess their surface morphology, size, and release kinetics. Particles were found to be porous in nature, as demonstrated by SEM (Figure 1A). Since CCL22-MPs are intended for local delivery, CCL22-MPs were manufactured to be 10–30 μm in diameter, a common size used for depot formulations to avoid phagocytosis and movement across biological barriers. More specifically, CCL22-MPs had a mean diameter of 17.4 μm and a polydisperse size distribution typically associated with homogenization-based emulsification methods (Figure 1B). CCL22-MPs exhibited some initial burst release, followed by a lag phase of approximately 8 days, which then transitioned into a more linear release (Figure 1C). Importantly, the CCL22 released from these MPs retained its biological activity compared to fresh, unencapsulated CCL22, demonstrating that this manufacturing method does not destabilize or harm the protein (Figure 1D). Heat-denatured CCL22 served as a negative control for the cell-based bioactivity assay, eliciting minimal response from the cells (Figure 1D). Traditional double emulsion CCL22-MPs served as a point of comparison for MPs manufactured by single emulsion methods.

### 3.2. Single Emulsion Encapsulation of CCL22

**Figure 2:**
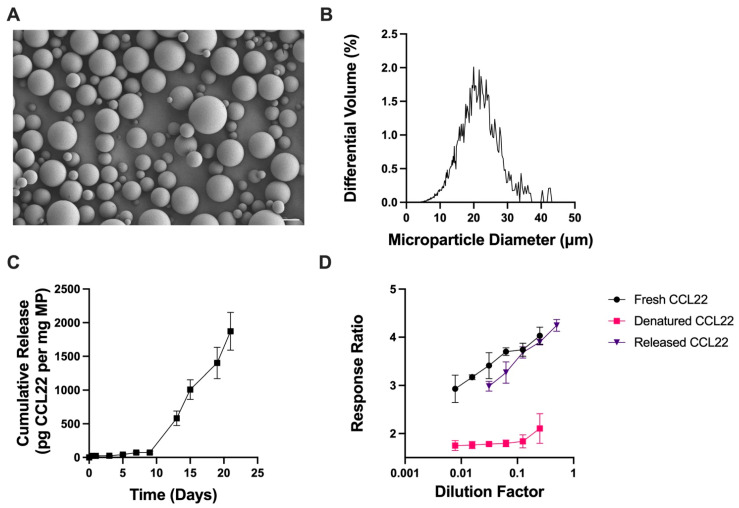
Characterization of single emulsion CCL22 microparticles. (**A**) Representative SEM image demonstrating smooth spherical MPs. (**B**) Size distribution as determined by volume impedance measurements (mean diameter: 20.2 ± 6.0 μm). (**C**) Release kinetics of CCL22 over a 21-day period, as determined via in vitro release assay and ELISA. Data are mean ± SEM (*n* = 3). (**D**) Biological activity of released CCL22 compared to fresh and denatured CCL22 through use of a cell-based assay. Results are mean ± SD (*n* = 3).

CCL22-MPs were then manufactured using a simpler homogenization-based, single emulsion (SE) solvent evaporation method, which is typically used to encapsulate hydrophobic, small-molecule drugs. CCL22-MPs formulated with the same 10 kDa MW PLGA-COOH polymer as before, but now using the SE method, were characterized similarly to assess their surface morphology, size, and release kinetics. As would be expected, SE CCL22-MPs exhibited differences in surface morphology and release kinetics compared to the DE formulation [31]. Specifically, SE CCL22-MPs had a smooth surface morphology with limited presence of pores or dimples in the surface (Figure 2A), while DE CCL22-MPs were porous in nature (Figure 1A). The observed differences in particle morphology can be attributed to the use of a single vs. two-emulsion phase system [31]. In particular, a DE two-phase system allows for the addition of osmolytes (in this case NaCl) to the inner aqueous phase, which contributes to MP surface porosity and also increases the initial burst release [29]. The size distribution for the SE CCL22-MPs was similar to that for the DE MPs, with a mean diameter of 20.2 ± 6.0 μm (Figure 2B).

Also consistent with previous reports, the DE CCL22-MPs had a noticeably higher initial burst release than the SE CCL22-MPs (Figure 1C and Figure 2C) [29,31]. After the initial burst, both DE and SE MPs exhibited a lag phase up until day 8, followed by a secondary burst phase and near-linear release. Both DE and SE CCL22-MPs released similar amounts of CCL22 at the end of the 21-day period. Differences in early release kinetics can be attributed to the surface morphology/internal microstructure, loading, and/or distribution of protein within the polymer matrix [32,33]. The porous outer structure of DE CCL22-MPs would also enable faster water penetration and less physical barriers to release CCL22 near the surface of the MPs, thus increasing the initial burst from these MPs. A lack of pre-established pores in the SE CCL22-MPs would necessarily result in relatively slower water penetration, which would subsequently reduce the rate of release of CCL22. These differences in surface porosity could thus be responsible for some of the differences in release kinetics between the two formulations [29].

For the SE method, CCL22 protein was first dissolved in DMSO, a polar aprotic solvent that is miscible with the dichloromethane (DCM) organic phase containing PLGA. While DMSO is commonly used to dissolve peptides and small-molecule drugs, it may impact the structure and stability of some proteins, potentially leading to destabilization and denaturation [15,16,17]. Although CCL22 released from SE MPs was successfully detected by ELISA antibodies, suggesting the corresponding epitopes remained intact throughout the encapsulation and release processes, it was still pertinent to determine whether the exposure of CCL22 to DMSO and DCM during the SE encapsulation method altered the biological activity of encapsulated CCL22. Importantly, CCL22 released from the SE MPs exhibited similar biological activity when compared to fresh CCL22. This is further supported by heat-denatured CCL22 exhibiting no dose response. The recovery of bioactive CCL22 after encapsulation via the SE method and then 14 days of in vitro release suggests that the manufacturing process adequately stabilized the encapsulated CCL22 protein without substantial degradation.

### 3.3. Altering Initial Burst Release of Homogenization-Based Single Emulsion CCL22-MPs

Different MP parameters can be altered to impact release kinetics, particularly the initial burst release [32]. In the case of DE MPs, a previous study demonstrated how altering the osmotic gradient between the inner and outer aqueous phases influenced burst release and overall release kinetics [29]. However, the lack of an inner aqueous phase in SE MPs limits the ability to alter the osmotic gradient as a method for modulating burst release and subsequent release kinetics. For SE MPs, different parameters may need to be altered to achieve some degree of initial burst when desirable.

Two parameters that can be tuned to alter the burst release of SE MPs are particle size and polymer–drug interactions. Previous studies have demonstrated that both particle size and polymer–drug interactions can influence release kinetics [14,34,35]. MP size may affect the length of the initial burst as smaller MPs have a greater surface area to volume ratio, leading to faster diffusion of the drug and potentially leading to a higher initial burst release. Small CCL22-MPs (12 μm) resulted in a greater burst release (Supplemental Figure S2). However, both small (12 μm) and large (23 μm) CCL22-MPs exhibited similar lengths of the lag phase and released similar amounts of CCL22 by the end of the 21-day period. This is consistent with the prior studies showing that changing the MP size can influence the initial burst release, thus enabling tunability of the SE CCL22-MPs.

Another relevant variable to assess is polymer–drug interactions. In the case of proteins, electrostatic interactions between the polymer and encapsulate have previously shown to play a role in governing the release from polymeric MPs [35]. CCL22, a positively charged protein, can interact with negatively charged carboxylate (COO^−^) end groups of PLGA, which can impede release. It was hypothesized that using hydroxyl (OH)-terminated PLGA instead would decrease electrostatic interactions, especially at earlier time points before carboxylate groups are introduced during polymer degradation by ester hydrolysis. The initial reduction in electrostatic interactions could lead to a shorter lag phase.

**Figure 3:**
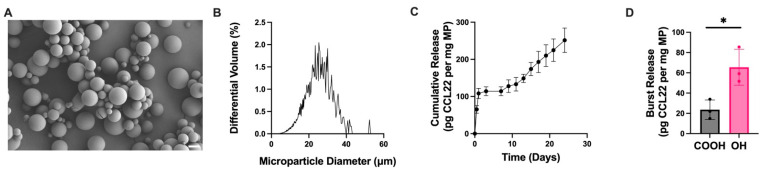
Altering initial burst release through changing polymer endcap. (**A**) Representative SEM image of CCL22-MPs manufactured via SE method with hydroxyl-terminated PLGA, demonstrating smooth spherical particles. (**B**) Size distribution as determined via volume impedance measurements (mean diameter: 22.5 ± 7.3 μm). (**C**) Release kinetics of CCL22 over a 21-day period as determined by in vitro release assay and ELISA. Data are mean ± SEM (*n* = 3). (**D**) Comparison of initial burst release between CCL22-MPs manufactured with PLGA-COOH vs. PLGA-OH. Results are mean ± SD (*n* = 3). A significant difference was identified by a two-tailed independent *t*-test. * *p* < 0.05.

Single emulsion CCL22-MPs formulated using PLGA-OH exhibited an initial burst release 3× greater than acid-terminated CCL22-MPs (Figure 3D). This increase in initial burst could be attributed to the alteration of electrostatic interactions between the CCL22 and the polymer. Initially, the PLGA-OH may have decreased electrostatic interactions, leading to a higher burst release than that from PLGA-COOH. In addition to the increase in initial burst from PLGA-OH CCL22-MPs, the polymer endcap also impacted the overall release kinetics (Figure 3C). While PLGA-OH CCL22-MPs exhibited a similar lag phase to PLGA-COOH CCL22-MPs, the PLGA-OH MPs released less CCL22 in the secondary phase, subsequently leading to less CCL22 released at the end of the 21-day period. Differences in release of CCL22 from PLGA-OH and PLGA-COOH MPs suggest that electrostatic interactions may play a larger role in governing release kinetics in the initial release kinetics. Additionally, the observed reduction in CCL22 released from PLGA-OH MPs could be a result of differences in the degradation profiles of the polymers used. Altogether, these results demonstrate that different parameters can be altered in the manufacturing of SE MPs to provide more tunability for the desired application and release profile.

### 3.4. Microfluidic, Continuous Manufacturing of Single Emulsion CCL22 MPs

Due to the advantages of continuous manufacturing in scaling and translation, a microfluidic method to produce SE CCL22-MPs was explored. CCL22-MPs were manufactured utilizing the same 10kDa MW PLGA-COOH polymer as before. When using this system, the PLGA and CCL22 were dissolved in a DMSO/DCM co-solvent system and added into the organic, dispersed phase pump, while 2% PVA was placed in the aqueous, continuous phase pump. These solutions were pumped at continuous flow rates through a flow-focusing chip to generate droplets. Flow rates were adjusted to generate particles with an average size comparable to those manufactured by the homogenization method. MPs manufactured via microfluidics were characterized using methods previously described above.

**Figure 4:**
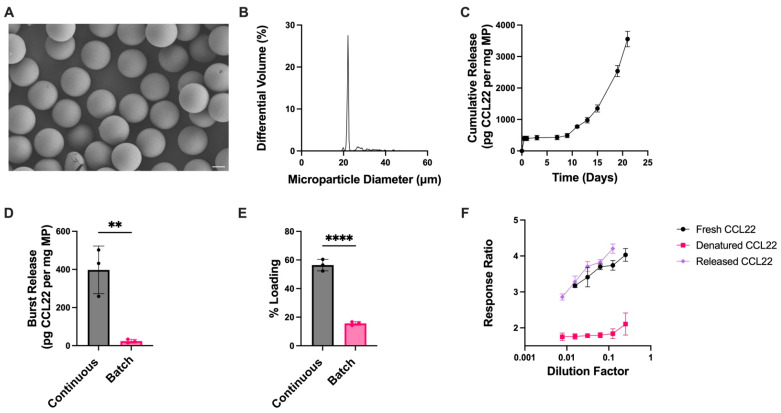
Microfluidic manufacturing of CCL22 microparticles. (**A**) Representative SEM image demonstrating monodispersed particles with smooth morphology. (**B**) Size distribution as determined via volume impedance measurements (mean diameter: 23.3 ± 4.1 μm). (**C**) Release kinetics of CCL22 over a 21-day period as determined by in vitro release assay and ELISA. Data are mean ± SEM (*n* = 3). (**D**) Burst release comparison between CCL22-MPs manufactured via microfluidic (continuous) and homogenization (batch) methods. (**E**) Comparison of protein loading between CCL22-MPs manufactured via microfluidic (continuous) and homogenization (batch) methods. Release and loading characterization results in C-E are presented as mean ± SD (*n* = 3). Significant differences in D–E were determined by a two-tailed independent *t*-test. ** *p* < 0.01, **** *p* < 0.0001. (**F**) Biological activity of released CCL22 compared to fresh (unencapsulated) and heat-denatured CCL22, measured via a cell-based assay. Results are mean ± SD (*n* = 3).

CCL22-MPs produced via microfluidics were monodisperse in size with a smooth surface morphology (Figure 4A,B). CCL22-MPs made via microfluidics had a significantly increased burst release compared to their counterparts made by a batch manufacturing process (Figure 4C,D). Previous work investigated differences in burst release from bupivacaine-loaded MPs manufactured via batch homogenization (polydisperse) compared to microfluidic-manufactured (monodisperse) MPs [36]. In that study, batch homogenization-manufactured MPs were found to have an increased burst release in comparison to their microfluidic counterpart. This was suggested to be due to the drug distribution within the polymer matrix; homogenization-based MPs resulted in the adsorption of bupivacaine towards the surface of the particles, while microfluidics allowed for more homogenous mixing of the drug within the polymer matrix. Interestingly, the results in this study showed opposite trends where monodispersed MPs had a greater initial burst compared to their homogenization counterparts. This difference could be at least partially attributed to the prior study utilizing a small molecule rather than a protein. In this case, the differences in polymer–drug interactions when the active ingredient is a protein with electrostatic charges could account for these differences, thus influencing release kinetics and potentially even the distribution of the therapeutic agent in the polymer matrix. Furthermore, this could be attributed (or exaggerated due) to differences in loading. MPs manufactured with microfluidics had significantly higher protein loading (Figure 4E). This is consistent with other reports that microfluidics may aid in increasing the encapsulation efficiency of different therapeutic agents [37].

While differences were seen in the initial burst release from microfluidic and homogenization MPs (Figure 4D), CCL22-MPs exhibited similar trends in release kinetics between the two manufacturing methods (Figure 2C and Figure 4C), and the biological activity of CCL22 from microfluidic-manufactured MPs was not adversely affected (Figure 4F). A comparison of the bioactivity assay cell response ratio between the MPs manufactured via different methods is shown in Supplemental Figure S3.

### 3.5. Broader Applicability: Encapsulation of a Second CCL(X) Chemokine CCL2

To further demonstrate the feasibility of this manufacturing method, a second chemokine previously used for therapeutic applications, recombinant CCL2, was encapsulated. Similarly to the aforementioned studies with CCL22-MPs, CCL2-MPs were manufactured using batch homogenization-based DE and SE methods. CCL2-MPs were also manufactured using the continuous, microfluidic-based SE method. All CCL2-MP formulations were characterized to assess surface morphology, size distributions, and release kinetics (Supplemental Figure S4). Similarly to CCL22, some differences in MP surface morphology and release kinetics were observed for the CCL2-MPs; however, all formulations successfully encapsulated and provided the sustained release of CCL2 that could be detected by ELISA. Collectively, these results with CCL2 suggest that more scalable SE methods and continuous processes also can be used to encapsulate other low-molecular-weight chemokines in PLGA MPs.

## 4. Conclusions

The feasibility of encapsulating low-molecular-weight chemokines using a single emulsion method was explored. Specifically, two chemokines, CCL22 and CCL2, that have a low number of alpha helices (one of the main components DMSO may impact) were successfully encapsulated in an SE method in both batch and continuous processes. This study also demonstrated the tunability of SE CCL22-MPs. In the case of DE protein-loaded MPs, altering the osmotic gradient, as well as other parameters, can impact release kinetics; however, some of these parameters are not applicable to SE MPs. Previous studies have demonstrated that altering particle size impacts drug release from PLGA MPs [38,39]. Smaller particles have a higher surface area to volume ratio allowing for greater water penetration, subsequently accelerating release [39]. Additionally, another study demonstrated that the negatively charged PLGA matrix interacts with positively charged peptides which impedes release, indicating that polymer–drug interactions may play a crucial role in governing the initial release [35]. To demonstrate that the tunable nature of polymeric MPs extends to these CCL22-MPs, particle size and polymer–drug interactions were modulated. Consistent with results from the aforementioned studies, smaller SE CCL22-MPs exhibited a greater initial burst release than larger SE CCL22-MPs. Furthermore, the release of a positively charged CCL22 protein from a PLGA-OH polymer matrix, with less negative charge density than PLGA-COOH, also accelerated the initial burst release, potentially by reducing protein–polymer electrostatic interactions. To further enable translatability and scalability [40], the SE batch process was adapted into a continuous process using microfluidics. CCL22-MPs generated using the microfluidic-based, continuous process exhibited differences in release kinetics when compared to those formulated using a batch process. In the future, methods employed with the batch process to modulate the release from SE CCL22-MPs could also be applied to MPs made using the microfluidic method.

With biologic therapies, ensuring their stability is crucial for enabling their translation. In the case of protein-loaded MPs, there is often concern of protein degradation and subsequent loss of biological activity attributed to manufacturing methods. As such, a DE technique has traditionally been employed to maximize protein stability and minimize loss of bioactivity. Here, we demonstrate that an SE homogenization batch method or continuous manufacturing microfluidic method encapsulating CCL22 leads to the retention of biological activity through the formulation process and 14 days of in vitro release. Furthermore, there were no differences in the biological activity of CCL22 released from DE MPs versus SE MPs. This suggests that protein stability and/or degradation is not negatively impacted by the SE manufacturing techniques, which are traditionally used to encapsulate hydrophobic, small-molecule drugs or peptides.

Future studies will be necessary to determine just how broadly applicable the SE manufacturing technique may be for encapsulation of other proteins with relatively low molecular weight and/or structural complexity, as well as long-term stability of SE MPs compared to DE MPs. While we cannot yet predict whether this approach will work for any given protein therapeutic, results from the current study could be applicable to the encapsulation of other low-molecular-weight proteins of interest in PLGA MPs using SE methods. This switch to continuous manufacturing (CM) techniques would have several advantages over batch processes. For instance, a recent analysis determined CM techniques have the potential to reduce both capital and operation expenses [41]. Furthermore, CM techniques can reduce the manufacturing footprint, enable easier optimization, and accelerate production times [42,43]. The switch to less complex and more scalable SE and CM methods could ultimately support scalable biomanufacturing for at least some sustained release biologic formulations.

## Data Availability

The raw data supporting the conclusions of this article will be made available by the authors upon request.

## Supplementary Materials

The following supporting information can be downloaded at: https://www.mdpi.com/article/10.3390/pharmaceutics17081056/s1, Figure S1: Differences in magnitude of initial burst release from CCL22-MPs manufactured via the DE and SE techniques; Figure S2: Influence of particle size on initial burst release from CCL22-MPs manufactured with PLGA-COOH; Figure S3: Comparison of biological activity across the three different manufacturing methods used to encapsulate CCL22 in PLGA-COOH; Figure S4: Encapsulation of additional low molecular weight chemokine CCL2.

## Abbreviations

The following abbreviations are used in this manuscript:

MPs	microparticles
SE	single emulsion
DE	double emulsion
CM	continuous manufacturing
PLGA	poly(lactic-co-glycolic acid)
