# Supplementary material for: Encapsulation of Therapeutic, Low-Molecular-Weight Chemokines Using a Single Emulsion, Microfluidic, Continuous Manufacturing Process
Source: Pharmaceutics. 2025 Aug 14;17(8):1056. doi: 10.3390/pharmaceutics17081056 (PMC12389030; doi:10.3390/pharmaceutics17081056)
Supplement: Supplementary file 1 [file pharmaceutics-17-01056-s001.zip › pharmaceutics-3779105-supplementary.pdf]

Supplemental Figures

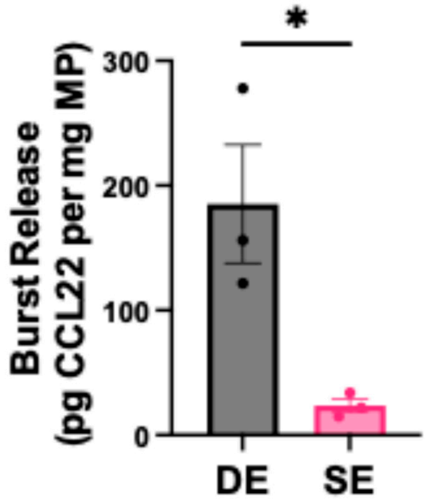

*Supplemental Figure S1: Differences in magnitude of initial burst release from CCL22-MPs manufactured via the DE and SE techniques. Data are presented as mean ± SEM (N = 3). A significant difference was identified by two-tailed independent t-test. * p < 0.05.*

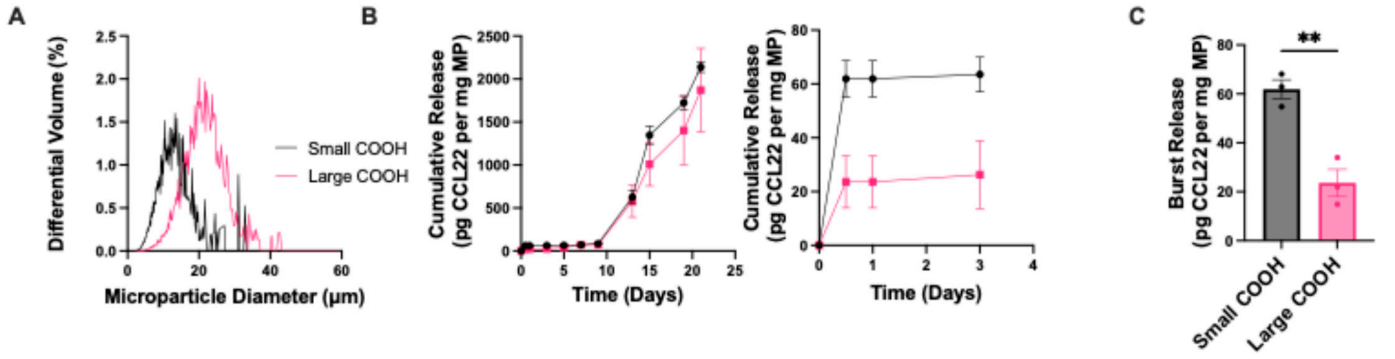

*Supplemental Figure S2: Influence of particle size on initial burst release from CCL22-MPs manufactured with PLGA-COOH. (A) Differences in size distributions between small and large CCL22-MPs (Small COOH mean diameter: 12.4 ± 5.1 µm; Large COOH mean diameter: 20.2 ± 6.0 µm). (B) Release kinetics of both small and large CCL22-MPs over a 21-day period, with a closer look at the initial 3 days of release as determined via in vitro release assays and ELISA. (C) Differences in initial burst release from both small and large CCL22-MPs. Data in (B-C) are presented as mean ± SEM (N = 3). A significant difference was identified by two-tailed independent t-test. ** p < 0.01.*

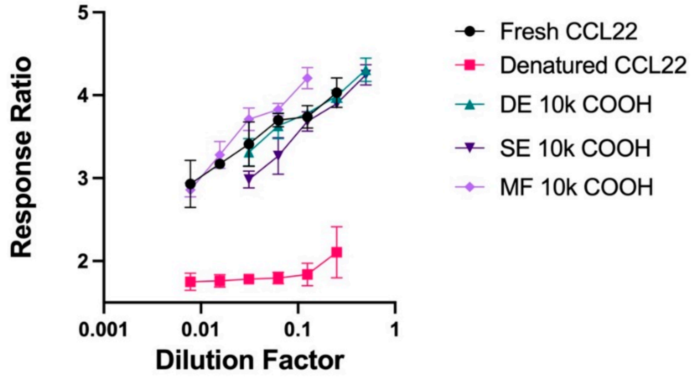

Supplemental Figure S3: Comparison of biological activity across the three different manufacturing methods used to encapsulate CCL22 in PLGA-COOH. Data represent mean ± SD (N = 3).

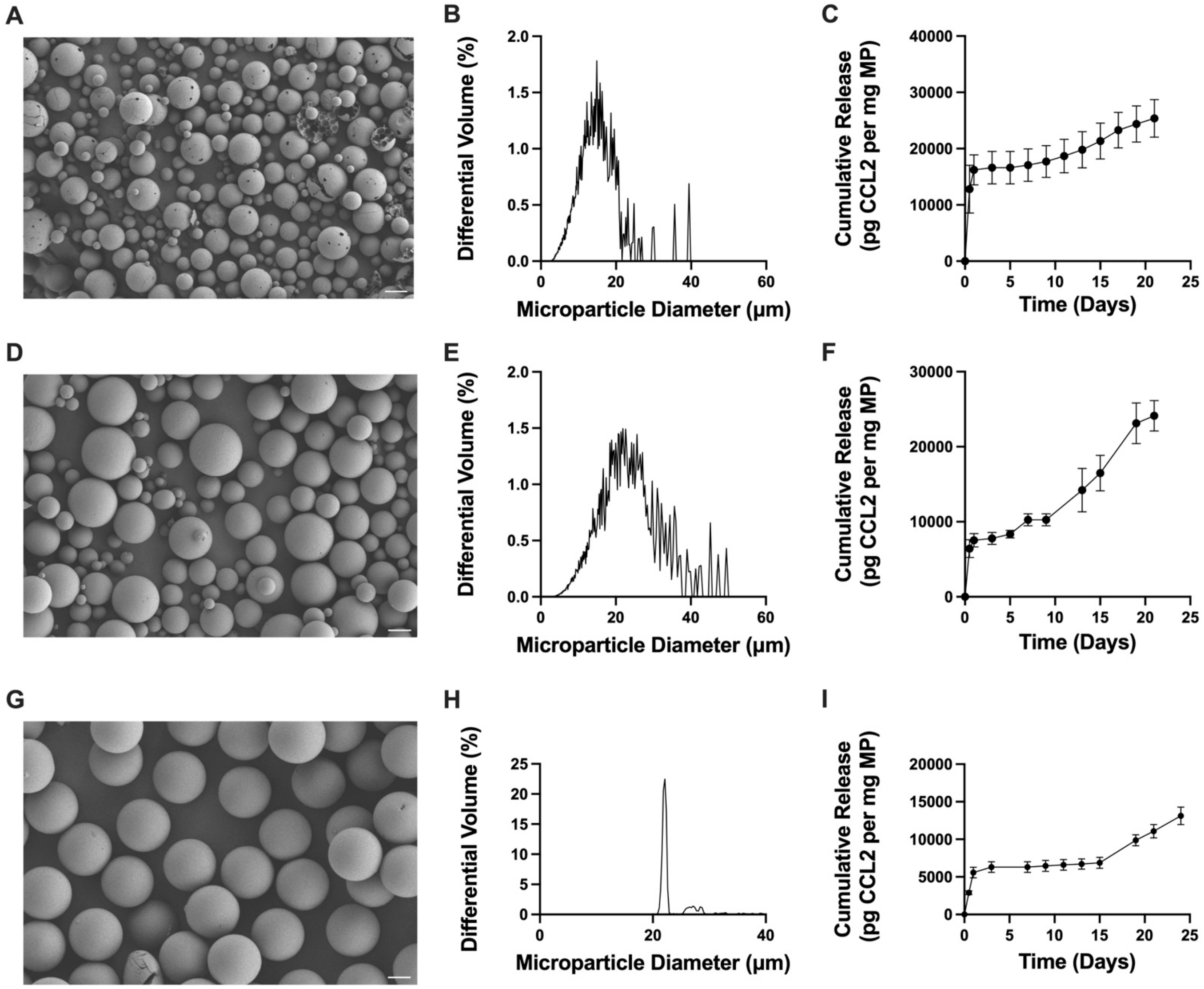

Supplemental Figure S4: Encapsulation of an additional low molecular weight chemokine CCL2. (A-C) Characterization of CCL2-MPs manufactured utilizing a DE technique. (A) Representative SEM image demonstrating porous spherical MPs. (B) Size distribution as determined via volume impedance measurements (mean diameter: 23.3 ± 8.1 µm). (C) Release kinetics of CCL2 over a 21-day period as determined via in vitro release assay and ELISA. (D-F) Characterization of CCL2-MPs manufactured utilizing a SE batch technique. (D) Representative SEM image demonstrating smooth spherical MPs. (E) Size distribution (mean diameter: 21.3 ± 8.1 µm). (F) Release kinetics of CCL2 over a 21-day period. (G-I) Characterization of CCL2-MPs manufactured utilizing SE and microfluidics. (G) Representative SEM image demonstrating monodispersed, smooth, spherical microparticles. (H) Size distribution (mean diameter: 23.5 ± 4.2 µm). (I) Release of CCL2 over a 21-day period.
